# Carbonylation of proteins—an element of plant ageing

**DOI:** 10.1007/s00425-020-03414-1

**Published:** 2020-07-01

**Authors:** K. Ciacka, M. Tymiński, A. Gniazdowska, U. Krasuska

**Affiliations:** Department of Plant Physiology, Institute of Biology, Warsaw University of Life Sciences-SGGW, Nowoursynowska 159, 02-776 Warsaw, Poland

**Keywords:** Carbonyl groups, Posttranslational protein modification, ROS, Senescence

## Abstract

**Main conclusion:**

**Carbonylation-ROS-dependent posttranslational modification of proteins-may be regarded as one of the important events in the process of ageing or senescence in plants.**

**Abstract:**

Ageing is the progressive process starting from seed development (plants) and birth (animals). The life-span of living organisms depends on many factors and stresses, which influence reactive oxygen species (ROS) level. The imbalance of their production and scavenging causes pathophysiological conditions that accelerate ageing. ROS modify nucleic acids, lipids, sugars and proteins. The level of carbonylated proteins can serve as an indicator of an oxidative cellular status. Several pathways of protein carbonylation, e.g. the conjugation with reactive carbonyl species, and/or a direct metal-catalysed oxidative attack on amino acids residues are known. Dysfunctional carbonylated proteins are more prone to degradation or form aggregates when the proteolytic machinery is inhibited, as observed in ageing. Protein carbonylation may contribute to formation of organelle-specific signal and to the control of protein quality. Carbonylated proteins are formed during the whole plant life; nevertheless, accelerated ageing stimulates the accumulation of carbonyl derivatives. In the medicine-related literature, concerned ageing and ROS-mediated protein modifications, this topic is extensively analysed, in comparison to the plant science. In plant science, ageing and senescence are considered to describe slightly different processes (physiological events). However, senescence (Latin: *senēscere*) means “to grow old”. This review describes the correlation of protein carbonylation level to ageing or/and senescence in plants. Comparing data from the area of plant and animal research, it is assumed that some basic mechanism of time-dependent alterations in the cellular biochemical processes are common and the protein carbonylation is one of the important causes of ageing.

## Ageing: still unknown fate in plants *versus* animals

Increased levels of oxygen in the Earth’s atmosphere allowed the evolution of animals and plants. However, the other side of the oxygen molecule “face” is the ability to accelerate oxidation reactions linked to ageing. The majority of ageing research concerns the eukaryotic cells, and some biochemical processes are common. Thus, the basic biological mechanisms of ageing at this level may be similar for plants and animals. Nevertheless, there is a discussion how to precisely define the term “ageing”. In animal science, ageing is not so simple to define because of (among others) the diversity of life forms-there are short-lived and long-lived organisms (Cohen 2018 and citations therein). Long-lived individuals and clonal organisms exist in the plant kingdom. Asexual reproduction of some plant species leads to formation of clones which may proliferate to form community-sized “individuals” of unusual longevity. As was demonstrated for *Lomatia tasmanica* (W.M. Curtis), longevity can be extended even to 40,000 years (Lynch et al. 1998). Long-lived organisms can be found in Spermatophyta. The maximum observed life-span of bristlecone pine (*Pinus longaeva* D.K. Bailey) is estimated to 4,600 years, giant sequoia (*Sequoiadendron giganteum* (Lindl.) J. Buchh.) to 3200 years, common juniper (*Juniperus communis* L.) to 2000 years, scots pine (*Pinus sylvestris* L*.*) to 500 years and apple (*Malus domestica* Borkh.) or English ivy (*Hedera helix* L*.*) to 200 years (Thomas 2013 and citations therein).

The term “program” adopted from computer science is commonly used in expressions linked to the physiology of living organisms. Terminology: “senescence program”, “programmed ageing” and “programmed death” potentially explain life-span fate (Thomas 2013 and citations therein). Nevertheless, ageing of an individual organism (considered as a whole organism) is rather a side effect of biological processes than a “programmed” event, with some exceptions when “programmed ageing” and eventually death occur, e.g. in the modules of colonial marine invertebrate *Botryllus schlosseri* (Cohen 2018 and citations therein). On the other hand, as discussed below, in plants “programmed senescence” of special cells or whole organs is an important developmental phase.

**Fig. 1:**
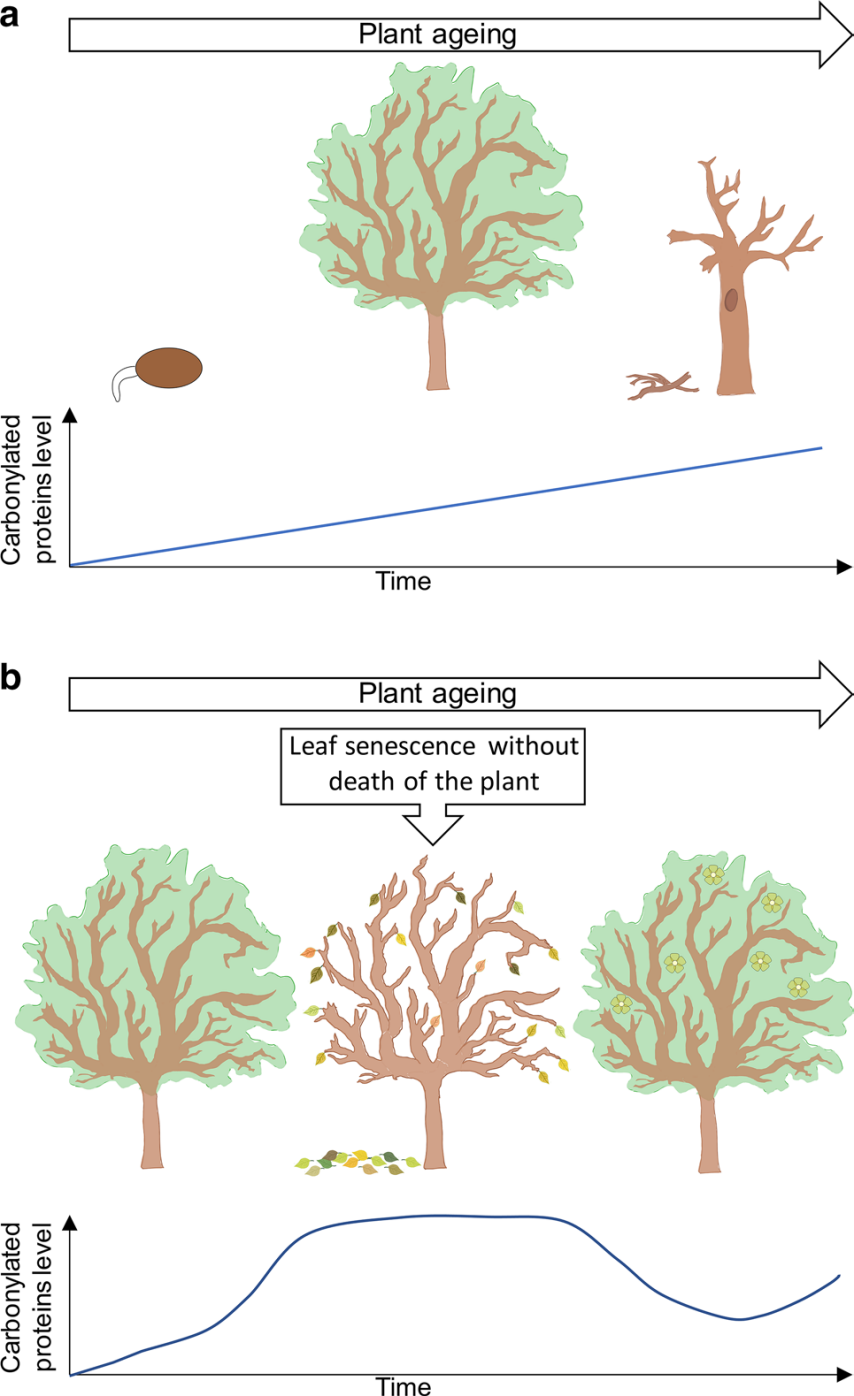
Changes in the level of carbonylated proteins during the life of a tree. **a** The progression of plant ageing. The highest level of the proteins marked with CO groups is achieved at last part of life-span (internal and external symptoms of tissues senescence). **b** Seasonal, environmental-dependent changes. Leaf senescence is not linked to ageing of the whole plant organism (the leafless tree) and is accompanied by an increase of carbonylated proteins level. The lowest content of oxidized proteins is achieved before the production of the offspring (the blooming tree)

The term “mortality” refers to a chance of death at a given age, while the term “life-span” describes a maximal life expectancy at birth (Thomas 2013). Senescence as a significant process is under the control of internal agents: hormones, signalling molecules and transcription factors. Their action depends on the stage of ontogeny or/and is stimulated by environmental factors (Lim et al. 2007; Thomas 2013). Plant scientists define the senescence as an evolutionary adaptation and highlight its physiological role in plant growth, development, reproduction and survival (Lim et al. 2007). Thus, senescence by definition is a phase of development that is a transdifferentiation event following the completion of growth, that may or may not be completed with death, and that is categorically dependent on cell viability and the expression of specific genes (Fig. 1) (Thomas 2013 and citations therein). Plant adaptation to the environment is accompanied by alterations of tissue structure and function. Senescence (e.g. programmed cell death) takes part in these modifications specifying cell fate (Gunawardena 2008). Therefore, senescence is believed to be a "programmed suicide" which allows plants to control their viability and integrity during the life cycle, the phenomenon also called Samurai Law of Biology (“it is better to die than to be wrong”) (Thomas 2013). In turn, the term “ageing” per se refers to the time-dependent changes of a living organism from birth through maturity, senescence and death. Both, senescence and ageing are linked to the deterioration processes. Additionally it has been proposed that senescence is a process of accelerated ageing (Thomas 2013).

Plant life strategy comprises the controlled death and the disposal of cells. Contrary to animals ageing of plants differs due to the seasonal cycle (Fig. 1) and persistence of autotrophs (Thomas 2002, 2013). Furthermore, in plants senescence and organ/tissue elimination are imprinted into programmed ontogeny phases and related to the inner reallocation of resources. These changes are the part of developmental processes and are not linked to ageing per se (Thomas 2002). Due to specific anatomical features and performance of undisturbed transport of water and/or metabolites, perforations in some tissues of plants are necessary. The selective cell death is the important physiological mechanism for the creation of structures with holes (e.g. xylem or aerenchyma), therefore plants evolved controlled autolysis (Moriyasu 1995; Thomas 2002). A self-destructive (monocarpic) senescence is a part of the life cycle of annual plants (e.g. sunflower (*Helianthus annuus* L.) or pea (*Pisum sativum* L.)). In this case only the seeds survive as viable structures (Sadras et al. 2000). Removing flower buds is a method to extend longevity of monocarpic plants. Nevertheless, this is not the rule. In most cereals the prevention of seed formation can even accelerate the senescence of the plant (Thomas 2002). Thus, plant life-forms which determine life-span (therefore ageing) distinguish plants from animals.

Mortality means that what begins comes to the end, and ageing is the process leading eventually to cell or organ death. Mechanisms of ageing are still not fully known and understood, therefore extensively studied, especially in modern societies dealing with an ageing population. The same trend is observed for plants (Thomas 2002; Höhn et al. 2017). Slower ageing (longer life-span) is exhibited for those species that produce greater seed mass, long-lived leaves or dense wood, which is related to higher survival elasticity (Adler et al. 2014; Munné-Bosch 2015).

The basic causes of ageing in animals are linked not only to oxidative stress, inflammation, mitochondrial dysfunction and accumulation of misfolded proteins (loss of proteostasis) but also to genomic instability, telomere shortening or/and attrition, epigenetic alterations, and modified intercellular communication (Cohen 2018 and citations therein). These processes lead to the impairment of cellular homeostasis and are characterized by the time-dependent persistent alteration of the functionality of cells and organs. An accumulation of cellular damage, in turn, enhances susceptibility to negative factors (Höhn et al. 2017). It is also accompanied by protein functional malformations linked to DNA damage. The results of many experiments conducted mainly on animal tissues discovered special markers related to the morphological and physiological alterations: development of enlarged nuclei or/and an elevated ageing-associated β-galactosidase activity (SA-β-Gal). Moreover, it has been demonstrated that time-dependent cellular disintegration is linked to the increase of reactive oxygen species (ROS) content (Ben-Porath and Weinberg 2005; Höhn et al. 2017 and citations therein).

Plants as less mobile organisms than animals are more prone to different environmental factors that are potentially mutagenic. Despite the rather high frequency of DNA damage under standard conditions, the mutation rate in plant cells is very low (Nisa et al. 2019 and citations therein). It means that, plants have to develop many protective systems. As they possess chloroplasts, it also seems that they have more complex DNA repair mechanisms compared to mammals (Ferrando et al. 2019 and citations therein). Like in animal cells, in plants the activity of enzymes of base excision repair pathway (BER) was identified. In mitochondria of potato (*Solanum tuberosum* L.) tubers under hypoxia conditions, the activities of apurinic/apyrimidinic endonuclease and uracil DNA glycosylase (the enzymes of BER) were significantly increased. However, under optimal assay conditions, the mechanism of DNA repair in mitochondria in potato tuber was not so efficient as in mouse liver (Ferrando et al. 2019). It can be assumed that the accumulation of DNA mutation is not the main cause of ageing in plants as very old bristlecone pine trees produce seeds with undiminished vitality, which germinate as well as seeds from younger trees (Lanner and Connor 2001).

In plants, senescence is defined as a complex deterioration process which can finally end in death of the whole organism or a single organ (Fig. 1). Factors that regulate this process are divided into internal (age, reproductive stage, a level of regulators of growth and development) and external (environmental signals and stressors) (Gan and Amasino 1997). In some cases, senescence can be reversible, e.g. the gerontoplast redifferentiation during strong tobacco (*Nicotiana rustica* L.) regreening (Zavaleta-Mancera et al. 1999).

Senescence of a leaf is accompanied by an intensive protein degradation to remobilize nitrogen to other parts (the sinks) of the plant. This proteolysis is highly regulated, and it has been proposed that proteins which are subjected to degradation are specifically marked by posttranslational modification, e.g. by carbonylation, depending on ROS reactivity.

“The Free Radical Theory of Ageing” was proposed by Denham Harman in 1956. Since that time, it is obvious that oxidative stress is an intrinsic element of ageing in animals and humans. The reactive oxygen-based cell death theory is commonly accepted also among plant scientists (Van Breusegem and Dat 2006).

### ROS participation in ageing

The permanent presence of oxygen in Earth’s atmosphere and its incomplete reduction or excitation is the main reason for ROS formation in cells (Mittler 2017). As was demonstrated for human fibroblasts the growth under high (40–50%) ambient oxygen concentration resulted in premature ageing. Contrary, an extended life-span of the same-type cells was observed under low (2–3%) oxygen content (Ben-Porath and Weinberg 2005 and citations therein). As for plants, the exposure of apple fruits to high oxygen (100%) level resulted in accelerated senescence (Qin et al. 2009).

The physiological function of ROS depends on the concentration. High content of these molecules leads to oxidative stress and eventually ends in death. By contrast, ROS at lower levels are key elements of signalling cascades, which are known to modulate the activity of mitogen activated protein (MAP) kinases (Rentel et al. 2004). Extended periods of oxidative stress linked to elevated ROS concentration are commonly accepted as significant stimulators of senescence progression (Colavitti and Finkel 2005). Furthermore, hydrogen peroxide (H_2_O_2_) is thought as the main ageing inducer. This consideration comes from data indicating that H_2_O_2_ treatment or inhibition of ROS-scavenging enzymes leads to premature senescence of cells (Ben-Porath and Weinberg 2005 and citations therein).

The ROS family include superoxide anion (O_2_^·−^), hydroxyl radical (^·^OH), H_2_O_2_, as well as peroxyl (ROO^·^), alkoxyl (RO^·^) and hydroperoxyl (HO_2_^·^) radicals (Demidchik 2015). ROS toxicity is derived from the ability to react with the important, cellular molecules: nucleic acids, proteins, lipids and sugars (Møller et al. 2007; Møller and Sweetlove 2010; Demidchik 2015). Highly reactive species (radicals) react with amino acids, peptides and proteins via various reactions: hydrogen abstraction, electron transfer (oxidation or reduction), addition, fragmentation and rearrangement, dimerization, disproportionation and substitution. ROO^·^ are involved in multiple reactions leading to formation of protein carbonyls (Davies 2016). Direct reactivity with cellular macromolecules was confirmed for both singlet oxygen (^1^O_2_) and ^·^OH. ROS overproduction together with dysfunctional antioxidant machinery is one of the main reasons for pathophysiological state of an organism resulting in death.

In plants, ROS are generated in mitochondria, peroxisomes, plastids (mostly chloroplasts) and in the apoplastic space (Corpas et al. 2015). In animals, including humans, it was demonstrated that the mitochondrial dysfunction was linked to high ROS production, and thus, it is suggested to be the main cause of ageing (Passos et al. 2007; Barja 2014). Mitochondrial damage or disorder is connected to electron leakage and generation of O_2_^·−^ as by-products, observed mainly on the complex I (NADH dehydrogenase) and complex III (cytochrome *bc*1 complex) (Fisher-Wellman and Neufer 2012). It has been demonstrated that cellular rest stage or ageing is linked to the increased macromolecules oxidation. In yeast that undergo cells growth arrest, mitochondria generate more ROS, which is accompanied by the enhanced protein carbonylation. Moreover, oxidative damage in the cell is directly related to the redox status of the quinone pool (Aguilaniu et al. 2001). Carbonylated proteins of mitochondrial origin were also detected in aged WI-38 human embryonic fibroblasts (Ahmed et al. 2010). The presence of proteins with carbonyl groups was also confirmed in mitochondria of senescent apple fruits. In addition, after accelerated ageing, fruit exposure to high oxygen concentration the level of such modified proteins increased (Qin et al. 2009). The proper cell metabolism is correlated with standard activity of Krebs’ cycle enzymes, which also undergo carbonylation (Kristensen et al. 2004; Meany et al. 2007). It can be supposed that carbonylation of mitochondrial proteins may interrupt the whole cell physiology and may be linked to a progressive ageing (Stadtman 2006). These findings indicate the strong implication of mitochondria in senescence accelerated especially under the conditions of oxidative imbalance.

Huge amounts of ROS are generated during oxidative reaction carried out in peroxisomes (del Río and López-Huertas 2016), which may lead to protein carbonylation. As it was demonstrated for proteins isolated from peroxisomes of castor bean (*Ricinus communis* L.), endosperm subjected to metal-catalysed oxidation (MCO) with CuCl_2_/ascorbate carbonyl groups were detected in malate synthase, isocitrate lyase and catalase (Nguyen and Donaldson 2005). Moreover, carbonylation of these proteins was linked to activity loss pointing on the vulnerability of peroxisomal proteins to oxidative damage (Nguyen and Donaldson 2005).

Fenton and Haber–Weiss reactions are tightly related to ROS production. The Fenton reaction depends on the constant presence of reductants, transition metal ions and H_2_O_2_. During this reaction ^·^OH is generated. This molecule is also produced throughout peroxidase-mediated Haber–Weiss reactions (Müller et al. 2009; Demidchik 2015). ^·^OH reacts only with molecules that are very close (a few nm of approximate diffusion radius), while H_2_O_2_ has longer half-life and can diffuse across membranes (Møller et al. 2011; Demidchik 2015; Jeevan Kumar et al. 2015; Davies 2016), thus considered to be the primary (direct or non-direct) cellular messenger (Møller et al. 2007).

One of the most important enzymatic producers of ROS is a plasma membrane respiratory burst oxidase homolog (Rboh)––a nicotinamide adenine dinucleotide phosphate (NADPH) oxidase (Jeevan Kumar et al. 2015). It has been proposed that NADPH oxidases in plants participate in regulation of cell death. Old leaves of *RBOHF2*-silenced barley (*Hordeum vulgare* L.) mutants exhibited increased leaf-tip necrosis and higher accumulation of salicylic acid (Torres et al. 2017).

The maintenance of optimal ROS concentration is based on the action of antioxidant enzymatic and non-enzymatic systems. The elementary enzymes involved in ROS modulation are as follows: various isoforms of superoxide dismutases (SODs), catalase (CAT), ascorbate peroxidase (APX), glutathione peroxidase-like (GPX-like) and glutathione reductase (GR). Thioredoxins, glutaredoxins and peroxiredoxins are also ROS scavengers (Demidchik 2015; Morscher et al. 2015). Non-enzymatic modulators of ROS content are as follows: reduced form of ascorbic acid (ASA) and reduced form of glutathione (GSH), proline, (Signorelli et al. 2014) carotenoids and α-tocopherols (Kranner et al. 2006; Demidchik 2015; Morscher et al. 2015). As was mentioned, SODs are the primary enzymatic antioxidants which catalyse the conversion of O_2_^·−^ into H_2_O_2_ (Alscher 2002). Further, CAT catalyses the change of two molecules of H_2_O_2_ into water and O_2_ (Mhamdi et al. 2010). Transcript levels of *SOD* and *GR* decreased in aged pea seeds (Yao et al. 2012). Additionally, accelerated ageing had a negative impact on the de novo transcription of those genes. On the other hand, the controlled deterioration procedure (artificial ageing) did not affect the transcript level of *CAT* in pea embryonic axes (Yao et al. 2012). GPX (in plants are present selenium-lacking GPX-like proteins) activity results in scavenging of peroxides, especially phospholipid hydroperoxides (Navrot et al. 2006). This enzyme may serve as a membrane and storage lipid protector. An increase in GPX-like activity was noted in sunflower embryonic axes isolated from artificially aged embryos (Morscher et al. 2015). GPX-like proteins utilize GSH and convert it into the oxidized form (GSSG). GSH as well as the high GSH/GSSG ratio maintains redox state of the cells at the physiological level (Kranner et al. 2006; Demidchik 2015). GR is responsible for preservation of an optimal GSH/GSSG ratio by the reduction of GSSG to GSH in a reaction requiring NADPH. Increased GSSG content may point to ageing, e.g. in seeds with a viability loss (Kranner et al. 2006). A decrease in the size of the total glutathione pool, with the strong increase in GSSG content was demonstrated for dormant and non-dormant sunflower embryos subjected to controlled deterioration (Morscher et al. 2015). Additionally, a lower CAT activity during accelerated ageing under high O_2_ concentration was detected. Nevertheless, a constant activity of this enzyme was measured at ambient O_2_ level. CAT isolated from peroxisomes of castor beans endosperms was carbonylated as a result of metal-catalysed oxidation (MCO). This modification only partly inhibited CAT activity (Nguyen and Donaldson 2005). It is supposed that this enzyme may have some evolutionary adaptation to oxidative attack (Nguyen and Donaldson 2005). In turn, peroxidases of class III (POx) are haem-containing glycoproteins which are involved in both scavenging and production of ROS. In senescing 6-week-old Arabidopsis plants, the activity and inducibility of POx were higher, while SOD activity and inducibility decreased (Abarca et al. 2001).

## Protein carbonylation: friend or foe?

A proper metabolism depends on a precise function of proteins, and unsettled cellular proteome can lead to the dysfunction of the entire organism. ROS participate in protein oxidation which occurs via around 60 different paths, including carbonylation (Madian and Regnier 2010; Møller et al. 2011). The induction of protein oxidation includes MCO, oxidation-induced cleavage, amino acid oxidation and the conjugation of lipid peroxidation products (Cecarini et al. 2007 and citations therein). Thus, oxidative protein modifications lead to formation of intra- and inter-protein disulphides, *S*-sulphenylation, *S*-sulphinylation and *S*-sulphonylation. The formation of carbonylated proteins is one of the major products of protein oxidation (Cecarini et al. 2007 and citations therein; Rudyk and Eaton 2014).

As was demonstrated for humans, increased protein carbonylation perturbs cellular homeostasis, which leads to metabolic disorders, and carbonylated proteins serve as indicators of a cellular oxidative imbalance (Dalle-Donne et al. 2003). Carbonylation as incorporation of carbonyls into the molecule, applies to proteins, lipids and nucleic acids (Dalle-Donne et al. 2003, 2006). It is estimated that during ageing, starvation or disease of various organisms about 10% of the proteome is more prone to carbonylation (Levine 2002; Sohal 2002; Maisonneuve et al. 2009). Thus, a positive correlation between the increase in protein carbonylation content and ageing has been proposed (Levine and Stadtman 2001; Höhn et al. 2017).

As the actual level of carbonylated proteins is an indirect but stable marker of ROS content, the analytical practice is based on reliable methods for detection and estimation of this posttranslational modification. Assays that are commonly used for carbonyl (CO) groups measurement in proteins mostly depend on 2,4-dinitrophenylhydrazine (DNPH). This reagent is suitable for spectrophotometric protein quantity estimation or for immunodetection (DNPH-specific antibodies) for protein quantity and quality analyses. Furthermore, in the literature the data obtained after fluorescent hydroxylamine or fluorescein-5-thiosemicarbazide detection can be found. Other methods based on an incorporation of biotin hydrazide label at sites of protein carbonylation, followed by visualization with avidin-coupled techniques are more often used, especially in the aspect of further MS analyses (Møller et al. 2011 and citations therein; Havelund et al. 2017).

**Fig. 2:**
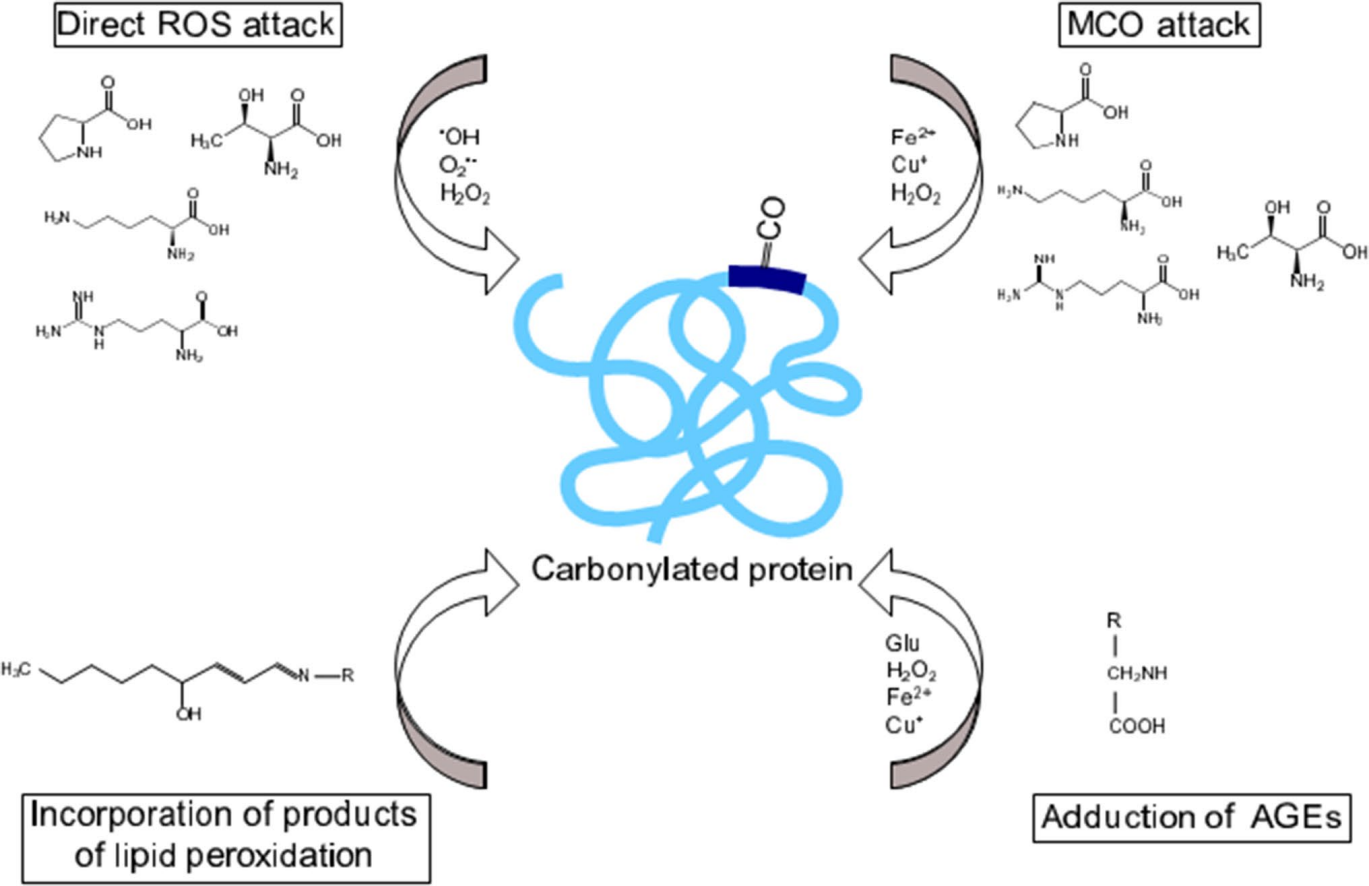
The most common pathways of protein carbonylation include direct ROS attack on amino acid residues (Pro, Arg, Lys and Thr), metal-catalysed oxidation (MCO) attack on Lys, Arg, Pro and Thr residues in the presence of ROS and reduced metal ions, adduction of advanced glycation end products (AGEs) formed in the presence of ROS, reduced metal ions and reducing sugars, e.g. glucose (Glu), and incorporation of products of lipid peroxidation, e.g. 4-hydroxynonenal. R represents amino acid residue of targeted protein for carbonylation

Protein carbonylation seems to be a selective process. Das et al. (2001) demonstrated that, during ageing of *Drosophila*, aconitase was the only mitochondrial protein characterized by the increased oxidation accompanied by the loss of its activity. The specificity of protein carbonylation was also demonstrated for *Escherichia coli* (Nyström 2005 and citations therein), yeast cells (Cabiscol et al. 2000), ageing flies (Yan et al. 1997; Sohal 2002) and plants (Johansson et al. 2004; Kristensen et al. 2004). Accumulation of carbonylated proteins is the result of even basic reactions (e.g. carbohydrate metabolism), protein maintenance and homeostasis as well as cellular motility as was shown for adult muscle stem cells (Baraibar et al. 2011). This modification occurs during whole ontogeny of living organisms, and some similarities in the pattern of this process can be found in plants and animals, but there is no strict correlation with the age of examined biological samples. The only comparisons between plants and animals concern this time in life-span when the offspring is generated connected with a low level of oxidative damage (Fig. 2) (Nyström 2005). The analyses performed on animals indicated that the content of carbonylated proteins increased starting from birth. Comparable alterations of the level of oxidized protein were observed in plants, e.g. Arabidopsis. Nevertheless, this high content of modified proteins decreased prior to the transition from the vegetative to reproductive phases (Fig. 1) (Johansson et al. 2004). Accumulation of oxidatively modified proteins, which are not inherited by daughter cells during cytokinesis, occurs during the replicative age of yeast (Aguilaniu et al. 2003). Moreover, the mother cells of the strain lacking the *sir2* gene (the silent information regulator) failed to retain modified proteins (Aguilaniu et al. 2003). Sirtuins or Sir2––NAD-dependent histone deacetylase––are life-span determinants, evolutionarily conserved from bacteria to humans. Thus, the results demonstrated by Aguilaniu et al. (2003) strongly indicate that the ability for keeping carbonylated proteins in the mother cells during division depends on the replicative age.

The first step in the oxidation of methionine (Met) and cysteine (Cys) is reversible in contrast to protein carbonylation. There is no strong proof regarding enzymatic or non-enzymatic nature of pathways to revert CO groups in amino acids residues (Dalle-Donne et al. 2003; Nyström 2005). Examination carried out on aged animals revealed the significant contribution of carbonylation in the regulation of proteins function and degradation (Levine 2002). Higher level of protein carbonylation was demonstrated to be linked with diseases such as Parkinson, Alzheimer, cancer, cataractogenesis, diabetes and sepsis (Levine 2002; Dalle-Donne et al. 2003). By contrast, the decrease in the level of proteins with CO groups was shown for skeletal muscle mitochondria of mouse with the prolonged life-span (Lass et al. 1998). The results of experiments carried out on several animal cells and tissues have indicated that the last third of life is accompanied by a strong increase in carbonylated protein content (Stadtman and Levine 2000). Moreover, during oxidative stress, disease or ageing the average level of proteins marked with CO groups increases and is estimated around one-third of the all molecules (Stadtman and Levine 2000). Some authors indicate that even half of all proteins are modified at the stage of ageing progression (Rao et al. 2018 and citations therein). On the other hand, prolonged life-span (reached by experimental manipulation) is linked to a lower content of carbonylated proteins, as was demonstrated using *Drosophila* flies (Levine and Stadtman 2001 and citations therein).

Ageing accompanied by a higher ROS level and an increased amount of oxidized proteins is also connected with the intracellular availability of free iron (Stadtman 1992; Stadtman and Levine 2000). Yeast mutants lacking YFH1p protein (the iron storage protein) showed higher carbonylation levels (Desmyter et al. 2004).

The correlation between loss of seed vigour and protein modifications was observed in seeds subjected to controlled deterioration (Rajjou et al. 2008). Thus, conditions which accelerate seed ageing favour the increase in protein carbonylation in Arabidopsis seeds (Rajjou et al. 2008), and apple embryos isolated from warm stratified seeds (Dębska et al. 2013). Prolonged warm stratification of apple seeds was accompanied by an increase in ROS accumulation, an elevated level of CO groups in extracts of soluble proteins isolated from embryonic axes, and resulted in a decrease in the germination rate of the embryos (Dębska et al. 2013).

In leaves of cereals (e.g. in wheat or barley) senescence plays a crucial role in crop productivity. As was demonstrated using fluorescein-5-thiosemicarbazide the increase in carbonylated protein level was observed during natural senescence of the wheat flag leaf (Havé et al. 2015). On the other hand, the authors also noted relatively high levels of carbonylated proteins in young expanding leaves. These findings correspond well to data for Arabidopsis (Johansson et al. 2004; Qiu et al. 2008) and maize (Prins et al. 2011).

The basic (typical) level of carbonylated proteins in mammalian cells reaches the value of around 1 nmol per mg of proteins. An increase up to 8 nmol per mg of proteins was detected in pathophysiological tissues (Dean et al. 1997). In plant cells the basic level of such modified proteins was estimated to around 4 nmol CO groups per mg of proteins (Romero-Puertas et al. 2002; Nguyen and Donaldson 2005). As the presence of carbonylated proteins during whole plant ontogeny was confirmed (Johansson et al. 2004), it has been proposed that some of ROS-modified proteins (or peptides) potentially can function as organelle-specific signals (Møller and Sweetlove 2010). Protein carbonylation is linked to the inhibition of enzymatic activity and even degradation of modified molecules (Levine 2002). This is particularly observed during ageing when the progressive decrease of proteolytic capacity and accumulation of proteins of lower catalytic activity occur (Levine and Stadtman 2001 and citation therein). Loss of protein function is especially observed when the modification concerns the active site. Nevertheless, till now there is no strong proof linked to the tight dependence of the amount of CO groups and the rate of activity inhibition (Levine 2002; Nguyen and Donaldson 2005).

The oxidation of carbohydrates or lipids [for example, formation of 4-hydroxynonenal (4-HNE)] leads to formation of reactive carbonyl species, which further can be added to the protein structure forming carbonyls (aldehydes or ketones) (Fig. 2). The results of in vitro experiments using mass spectrometric analysis indicate that around 99% of proteins modified by 4-HNE contained a free CO group (Bruenner et al. 1995). Lipid-derived aldehydes or ketones come from peroxidation and breakdown of polyunsaturated fatty acids (PUFAs), e.g. linoleic acid. Such products of lipid peroxidation are mobile, can diffuse across membranes and may covalently modify proteins which are localized far from the ROS generation site. It is even proposed that this mechanism of protein carbonylation is more widespread than direct oxidation of amino acid residues (Schneider et al. 2001; Yuan et al. 2007). There are various mechanisms of protein modification by reactive electrophilic lipid peroxidation products (oxoLPPs) (Griesser et al. 2017). Nucleophilic lysine (Lys) and arginine (Arg) residues can be modified by oxoLPPs, including saturated aldehydes (alkanals) and oxo-carboxylic acids, via mechanism of the Schiff base formation. Lys, Cys and histidine (His) residues can form Michael adducts with α,β-unsaturated aldehydes [(hydroxy-)alkenals, hydroxy-alkadienals and alkatrienals]. Formation of Michael adducts of protein-oxoLPPs with the carbonyl group shifts the carbonyl signal from the lipid to the protein fraction. Additionally, there are other (and more diverse) products of the reaction of dicarbonyls, e.g. glyoxyal or methylglyoxyal and protein-bound nucleophiles, e.g. carboxymethyl derivatives and hemiaminal adducts with Lys, His or Arg (Griesser et al. 2017).

CO groups are also formed by a metal-catalysed oxidative (MCO) attack (in the presence of reduced metal ions, e.g. Fe^2+^ or Cu^+^ and H_2_O_2_) on amino acids residues: asparagine (Asn), Lys, Arg, proline (Pro) or threonine (Thr) (Fig. 2) (Dalle-Donne et al. 2003; Møller et al. 2011). The transition metal ions are able to reduce H_2_O_2_ to ^·^OH which oxidizes amino acids residues in its immediate proximity (Berlett and Stadtman 1997). As was shown MCO attack is a site-specific process in which oxidation involves only one or a few amino acids at the metal-binding sites of the protein (Stadtman and Oliver 1991). The product of Pro and Arg carbonylation is glutamic semialdehyde, and for Lys modification-aminoadipic semialdehyde, a marker of protein damage (Nyström 2005; Møller et al. 2011; Davies 2016).

In the presence of reducing sugars, which can react with Lys and Arg, the formation of glycation products (Amadori and Heyns compounds) are observed. They are prone to ROS attack (Matamoros et al. 2018 and citations therein). As a result, advanced glycation end products (AGEs) are generated. The increase in AGEs accumulation is characteristic for aged human tissues, e.g. protein glycation targets are human lens proteins or collagen (Sajithlal et al. 1999; Smuda et al. 2015). MCO and free radicals are strongly involved in formation of AGEs (Fig. 2), and AGE-induced protein cross-linking as was demonstrated for collagen (Sajithlal et al. 1999). The age-dependent increase in plant protein glycation was confirmed in Arabidopsis leaves (Bilova et al. 2017) and aged bean (*Phaseolus vulgaris* L.) nodules (Matamoros et al. 2018).

Protein carbonylation is also the result of direct ROS reaction with Pro, Arg, Lys and Thr residues (Fig. 2); thus, the incorporation of reactive carbonyl derivatives into peptides by interaction with Cys, His and Lys, and the adduction of AGEs products or MCO attack are the main causes of the generation of this irreversible modification (Fig. 2) (Yan and Forster 2011; Møller et al. 2011).

**Fig. 3:**
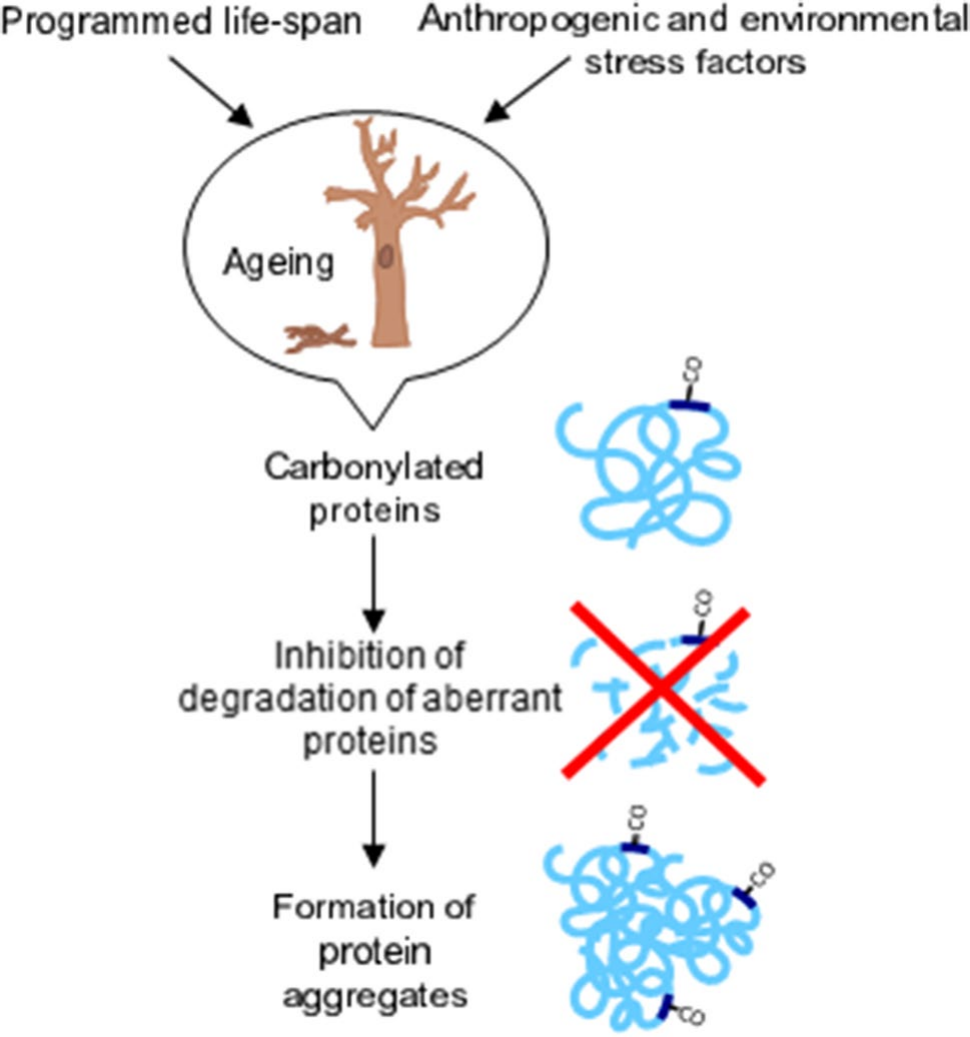
Ageing imprinted into the program of life-span or accelerated by anthropogenic and environmental stress factors is linked to the increase of carbonylated proteins level. The enhanced content of oxidized proteins may negatively implicate proteolytic machinery leading to the formation of proteins aggregates

Carbonylation of proteins initiates modification of their structure by the unfolding and exposure of the hydrophobic core, which is inside the folded molecule. These alterations allow interactions between oxidized proteins leading to formation of insoluble aggregates (Fig. 3) (Grune et al. 2004; Nyström 2005; Petrov and Zagrovic 2011). Furthermore, the CO group of one protein can react with an amino group of another molecule (Schiff base formation) stimulating the creation of aggregates. Such increase of aggregates content may occur without further oxidation (Höhn et al. 2011). Therefore, carbonylation may be considered as a positive process marking aberrant proteins even independently of direct ROS action. Hence, this modification would serve as an additional control of proteins quality (Dukan et al. 2000; Grune et al. 2004; Nyström 2005). Furthermore, under oxidative stress conditions, the system of the control of proteins quality, consisting of the various chaperones and ATP-dependent proteases, is responsible for removal of irreversibly damaged proteins (Smakowska et al. 2014 and citations therein). It is also proposed that dysfunctional proteins are more prone to oxidative alterations (Nyström 2005 and citations therein). Thus, carbonylation of abnormal proteins may serve as a signal for initiation of degradation instead of the chaperone/repair pathway. Thus, this “marking mechanism” ensures the exclusion of damaged proteins from metabolic pathways (Dukan et al. 2000). As was demonstrated for plants mitochondria, some chaperones and antioxidant enzymes may undergo carbonylation per se, especially under prolonged oxidative stress conditions, leading eventually to cell death (Smakowska et al. 2014 and citations therein). And again, it could be another important physiological function of proteins carbonylation maintaining the current program of growth and development (senescence and death of some cells), especially in plants.

Protein carbonylation seems to be not a random process, and several groups of researches have been working on the identification of the specific carbonylation sites (CS), mostly localized on the surface of the molecules (Maisonneuve et al. 2009; Höhn et al. 2017). MALDI-TOF and LC nano-ESI MS/MS techniques used to identify CS in oxidized bovine serum albumin (BSA) and some proteins from *Escherichia coli* led to development of the concept of the carbonylation "hot spots". The special amino acids sequence in BSA-Arg (R), Lys (K), Pro (P) and Thr (T) (the so-called RKPT-enriched regions)-is present and serves as the carbonylation target. Such CS located within RKPT-enriched regions were four times more prone to modification than those located outside the regions, and were potentially involved in selective protein carbonylation. Moreover, the close presence of iron binding sites with RKPT-enriched regions in proteins increased susceptibility to direct MCO attack (Maisonneuve et al. 2009). Not all RKPT-enriched regions underwent carbonylation; thus, the authors proposed that other mechanisms were also required for their specific modification (Maisonneuve et al. 2009).

The knowledge about the site of carbonylation in protein structure is available in CarbonylDB data base (https://digbio.missouri.edu/CarbonylDB/), a manually curetted resource (Rao et al. 2018).

## Turnover of carbonylated proteins

The induction or/and progression of senescence accompanied by an increase in carbonylated protein content are associated with disturbed proteostasis. Irreversibly modified (carbonylated) proteins should be degraded, otherwise they will form toxic, insoluble aggregates (Smakowska et al. 2014). Oxidized proteins are degraded by the proteasomal system-20S proteasome, which is ATP and ubiquitin independent. Additionally, some data indicated that during oxidative stress 26S proteasome (ATP/ubiquitin-dependent pathway) is inhibited (Shringarpure et al. 2001; Grune et al. 2003; Kästle et al. 2012). In sugar-deprived maize root tips, carbonylation of the 20S proteasome was connected with changes in the peptidic activities of 20S proteasome leading to the stimulation of chymotrypsin-like, peptidylglutamyl-peptide hydrolase and caseinolytic-specific activities and the inhibition of trypsin-like specific activity. These alterations in specific activities of proteasomes were similar to those observed after mild oxidative treatment (by MCO) of 20S proteasome purified from non-stressed tissue (Basset et al. 2002). For germinating apple embryos higher proteolytic activity was accompanied by a decrease of carbonylated proteins level. These data suggest the relationship between protein carbonylation rate and stimulation of protease activity (Krasuska et al. 2014). In Arabidopsis, two classes of chaperones and the inner membrane-embedded ATP-dependent metalloproteases (FTsH4) participate in the prevention of the accumulation of carbonylated proteins (Gibala et al. 2009; Smakowska et al. 2016). FTsH proteases have been identified in mammals and plants. A positive correlation between the age and the level of carbonylated mitochondrial proteins was demonstrated for Arabidopsis mutant lacking FTsH4, growing under short-day photoperiod (Gibala et al. 2009; Smakowska et al. 2014 and citations therein).

Ageing-dependent accumulation of carbonylated proteins results in the formation of protein aggregates as was first demonstrated in 1842 by Hannover for the cytosol of old neurons. These age-related protein aggregates are known as “lipofuscin” (Terman and Brunk 2004, 2006), “age fluorophore” or “age pigment” (Gutteridge 1984; Koistinaho et al. 1990). One of the possible explanations for the formation of protein aggregates may be the fact that degradation of oxidized proteins is overwhelmed (Castro et al. 2012). To support this hypothesis, it was demonstrated that part of carbonylated actin subjected to oxidative stress was degraded via the proteasome. However, inhibition of proteolysis leading to the formation of aggregates was dependent on the intensity or duration of the stimulus (Höhn et al. 2011; Castro et al. 2012). It was also proposed that the inhibition of proteasome concerns mostly postmitotic cells, as was demonstrated for neurons (Grune et al. 2004). Lipofuscins consist of highly oxidized crossed-linked molecules––proteins, lipids and sugars––and also can bind transition metal ions leading to formation of ROS via Fenton reaction. This makes lipofuscins an extra source of ROS. Furthermore, protein aggregates are able to change dynamics of gene expression (Catalgol et al. 2009; Kästle et al. 2012) and consequently are involved in the progression of ageing (Grune et al. 2004). Additionally, the autophagy/lysosomal degradation pathway was involved in the removal of oxidized proteins (Dunlop et al. 2009). Autophagy/lysosomal degradation of carbonylated proteins played a significant role at early stages of stress induction in rat cardiomyocytes under nitro-oxidative stress. By contrast, proteasomal degradation of carbonylated proteins was linked to the later time points of induced stress conditions (Griesser et al. 2017).

In plants the link between senescence, protein carbonylation and decreased ability to protein degradation (increased possibility of protein aggregates formation) was confirmed for bean in the context of the control of cell metabolism and nodule senescence (Matamoros et al. 2018 and citations therein), as well as in apple embryos subjected to artificial ageing (Dębska et al. 2013). Carbonylation led to aggregation of leghaemoglobin in bean nodules (Matamoros et al. 2018). Long-time (13 years) stored *orthodox* type seeds of beech (*Fagus sylvatica* L.) were characterized by low vitality related to the increased level of carbonylated proteins and carbonylation of proteins responsible for protein degradation. An impaired proteolytic machinery resulted in accumulation and formation of aggregates of proteins with carbonyl groups (Kalemba and Pukacka 2014). However, it is postulated that plants synthesize inhibitors of protein aggregation and these metabolites, in addition to their basic metabolic functions, could also suppress this unwanted process and participate in extension of plants longevity. These inhibitors of protein fibrillation (formation of insoluble ß-sheet-rich structures, which is a common phenomenon in patients suffering from diseases of lifestyle and ageing) are especially detected in long-lived plants (older than 100 years), although could be found also at lower concentrations in tissues of annual plants (e.g. herbs or/and spices) (Mohammad-Beigi et al. 2019). Therefore, synthesis of anti-aggregative compounds might be one of many features that potentially counteract the negative effect of protein modification (including carbonylation) and distinguish plants from animals.

## Conclusions

Ageing or senescence is accompanied by visible changes in the whole body (the animal or the plant) but mainly by physiological malformations, such as accumulation of aberrant proteins. The increased content of abnormal proteins alters proteome, thus results in dysfunction of the entire organism. Ageing of an organism is marked by the enhancement of protein carbonylation, and as was shown for animals, any prolongation of the life-span is linked to the lowering of oxidized protein levels. Incorporation of CO groups into proteins, lipids and nucleic acids occurs during intensive ROS production. Consequently, acceleration of ageing is connected with oxidative stress particularly under conditions of insufficient repair of cellular damage (Fig. 3). Moreover, an impaired protein degradation system is the cause of formation of protein aggregates. More and more has been revealed about life-span maintenance because science and medicine try to do everything to delay the inevitable. It seems therefore that understanding the carbonylation process can help long-lasting health and vitality for both plants and animals. It could be assumed that proteins carbonylation may play a key role in the senescence/ageing regulation. This protein modification may have dual effect depending on protein function and the level of proteins with CO groups.

Considering the fact that due to environmental pollution and anthropogenic activity the life-spans of plants are shortened; thus, more studies of the prevention of ageing are required. Despite the knowledge the authors have, some questions arise. Do they know all the pathways of protein carbonylation? Are there some other metabolites related to oxidation, and can they speed up ageing? Another important and unsolved problem concerning plants as a food source is the possibility that carbonylated proteins of plant origin negatively influence human health.

### *Author contribution statement*

Conceptualization: UK and KC. UK, AG and KC wrote the manuscript. Figures were prepared by MT. The final version of the manuscript and editing were done by UK, AG, KC and MT.
